# Real-World Evidence of the Impact of CanAssist Breast on Physician’s Decision About the Use of Adjuvant Chemotherapy in Early Breast Cancer

**DOI:** 10.7759/cureus.75622

**Published:** 2024-12-12

**Authors:** Somashekhar S P, Shekar Patil, Rajeev Kumar, Krishna Prasad, Devanhalli Govinda Vijay, Mandeep Singh Malhotra, Rohan Khandelwal, Ajay Bapna, Karthik S Udupa, DC Doval, Avinash C B, Kiran Shankar, Ananth Pai, Chaturbhuj Agrawal, Ravi Thippeswamy

**Affiliations:** 1 Surgical Oncology, Aster CMI Hospital, Bangalore, IND; 2 Medical Oncology, Healthcare Global Enterprises (HCG) Cancer Center, Bangalore, IND; 3 Surgical Oncology, Breast Services, Rajiv Gandhi Cancer Institute and Research Centre, New Delhi, IND; 4 Division of Medical Oncology, Department of Internal Medicine, Kasturba Medical College, Manipal Academy of Higher Education (MAHE), Mangalore, IND; 5 Surgical Oncology, Healthcare Global Enterprises (HCG) Aastha Cancer Centre, Ahmedabad, IND; 6 Surgical Oncology, C K Birla, Gurgoan, IND; 7 Breast Surgery, General Surgery, CK Birla Hospital, Gurugram, IND; 8 Medical Oncology, Bhagwan Mahaveer Cancer Hospital and Research Centre, Jaipur, IND; 9 Oncology, Kasturba Medical College of Manipal, Manipal, IND; 10 Medical Oncology, Rajiv Gandhi Cancer Institute and Research Centre, New Delhi, IND; 11 Medical Oncology, ClearMedi Healthcare, Mysuru, IND; 12 Surgical Oncology, Cauvery Heart and Multi-Specialty Hospital, Mysuru, IND; 13 Medical Oncology, Kasturba Medical College of Manipal, Manipal, IND

**Keywords:** canassist breast, early breast cancer, immunohistochemistry(ihc), india, prognostication

## Abstract

Background

Clinicians use prognostic biomarker/multi-gene-based tests for predicting recurrence in hormone receptor-positive/HER2-negative (HR+/HER2−) early-stage breast cancer (EBC). CanAssist Beast (CAB) uses the expression of five protein biomarkers in combination with tumor-specific parameters such as tumor size, histopathological grade, and lymph node status to predict the risk of distant recurrence within five years of diagnosis for patients with HR+/HER2−, EBC. The current study aimed to evaluate the impact of prognostic tests on adjuvant chemotherapy decisions by assessing the agreement between clinical and CAB risk stratification as low-risk (LR) or high-risk (HR) for distant recurrence.

Methods

The primary study group included 300 patients with HR+/HER2−, EBC diagnosed between 2016 and 2021. The clinical risk assessment and recommended treatment plan were captured before and after receiving the results for CAB. The risk stratification of patients into CAB LR and HR was obtained. Finally, compliance with CAB was analyzed by assessing the concordance of treatment prescribed with the CAB risk category.

Results

Before performing the CanAssist Breast test, patients were stratified based on clinicopathological features, with 52% of patients as LR, 21% as HR, and 27% of patients distributed as uncertain/intermediate risk (IR) category. CAB re-stratified the same cohort of patients, 67% as LR and 33% as HR, which was 15% higher than that of clinical LR assessment. The clinical IR category was re-stratified by CAB as 51% LR and 49% HR. Changes in treatment recommendations were seen in both clinical HR and clinical LR groups, which were 87% and 85%, respectively.

Conclusions

CAB has a significant impact on chemotherapy decisions. CAB provides definite treatment recommendations for patients with clinical intermediate risk. Overall, CAB has changed treatment recommendations in 23% of the cohort and for 88% of clinical IR patients helped physicians make a treatment decision.

## Introduction

The inclusion of chemotherapy in the treatment planning for HR+/HER2− EBC is judged by the risk of cancer recurrence. The clinical characteristics of a tumor, including its size, node status, histological grade, and proliferation markers (ER, PR, HER2, and Ki-67), are the primary factors used to assess the risk of cancer recurrence [1-5]. The use of tumor anatomical features to predict patient outcomes is important, but emerging evidence suggests that these features may not provide accurate or reliable prognostic information [6]. This suggests that other factors, such as those that are predictive of cancer recurrence, may be overlooked when relying solely on tumor anatomical features. Studies have shown that some node-negative (N0) patients require chemotherapy for a better prognosis, while some node-positive (N+) patients have a good prognosis without chemotherapy [7]. In fact, it has been reported that fewer patients with tumors < 2 cm benefit from chemotherapy [8,9]. This shows that prognosis based on clinical factors alone could lead to over/undertreatment.

Prognostic tests have addressed this issue with accurate prognostication and thereby led to optimum treatment recommendations [10-15]. Currently, widely used multigene tests use either microarray or RT-PCR techniques to arrive at a risk score predictive of distant recurrence. These tests have enormous data from prospective randomized trials or retrospective cohorts of randomized trials [13-15]. It is noteworthy that of the trial cohort, only 4.2% were comprised of Asian women; therefore, they are underrepresented in these trials. [16,17]. It would be of great value to showcase multi-gene tests that provide similar prognostic information across diverse cohorts [18]. These observations are of utmost importance for the general applicability of these tests to the global population and more so to Asian patients, due to underlying inherent differential racial factors between Asian and Caucasian women associated with breast cancer in addition to differences in clinical parameters, both of which could lead to the differential prognosis in these diverse populations [19,20].

CAB, a prognostic test, uses an immunohistochemistry platform to predict recurrence risk using a machine learning algorithm [21]. CAB has been validated on retrospective cohorts from India, the USA, and Europe, demonstrating similar prognostic ability across diverse cohorts [22-25]. CAB has shown its effectiveness in prognosticating EBC patients even in subgroups of patients aged below 50 years, node-positive patients, and patients with luminal-like characteristics [23,25].

CAB has been in clinical use since 2016 and has helped patients from South Asian countries plan their treatment [26]. In this current report, we describe the impact of CAB risk predictions on’ clinical decision-making and changes in decisions about chemotherapy use in EBC before and after the use of CAB.

## Materials and methods

Study population and design

This ongoing prospective study cohort consisted of 300 patients who were prescribed CAB across various hospitals in India between 2016 and 2021. This cohort included patients with HR+/HER2− EBC. The authors filled out two questionnaires before and after performing CAB. The first questionnaire is about ‘Clinical Risk Assessment’ (see below), which captured risk assessment-based clinicopathological parameters in the absence of a prognostic test. The second questionnaire captured the risk assessment per prognostic test (see below Prognostic Risk Assessment). The final treatment was decided by clinicians after the prognostic test, in this case, the CAB report.

Clinical risk assessment

Clinical risk assessment was done based on clinicopathological features such as tumor size, node status, histological grade, proliferation markers (Ki-67), and age. The opinion on the treatment plan as per clinical risk was categorized into three groups. i) Low risk: N0 tumors, low histological grade, elderly patients, and low Ki67 are considered to have a low risk of recurrence and were recommended not to take chemotherapy. ii) High risk: N+ tumors, grade 3 tumors, age ≤ 48 years at the time of diagnosis, and high Ki67 are considered to have a high risk of recurrence and were recommended to take chemotherapy. iii) Intermediate risk: those for whom the physicians were in a dilemma on using chemotherapy.

Prognostic risk assessment

CAB is a prognostic test for Stage I & II patients with hormone receptor-positive (ER/PR+) and Her2- breast cancer. CAB involves IHC analysis of five biomarkers (CD44, ABCC4, ABCC11, pan-cadherin, and N-cadherin). Using the IHC data and coupled with three clinicopathological parameters-tumor size, tumor grade, and node status, a proprietary machine learning algorithm generates a risk of breast cancer recurrence category as low-risk or high-risk.

CAB performance

After the physician referred the patient for CAB, FFPE tumor blocks were shipped to the OncoStem laboratory. Information on tumor characteristics, ER, PR, HER2−, and Ki-67 was as per the histopathology report shared with OncoStem. Blocks with >30% (based on hematoxylin and eosin staining) tumor content were processed for CAB. Immunohistochemistry of the five CAB biomarkers was performed on the automated Roche Benchmark XT machine, and the CAB risk score was obtained using a machine learning algorithm as described earlier [21,27].

Statistical analysis

In this study, Microsoft Excel (Redmond, USA) was used to create graphs for visualizing the data.

Ethical statement

For this study, only existing data from routine diagnostic procedures performed as part of routine patient care was used. Patient consent for the use of these data was obtained. Ethical approval was obtained from the Sri Venkateshwara Hospital Ethics Committee (ERC/298/Inst/KA/2013/RR-19). 

Preprints

This article was previously posted to the Research Square preprint server on 18 May 2021.

## Results

Baseline characteristics of the patients

Out of the 300-patient cohort, all were women. The median age of diagnosis was 59 years (26-81). 22% were aged below or equal to 48 years, and 78% were above 48 years. 33% of patients had T1 (≤2 cm), 63% had T2 (≥2.1 cm), and 4% were T3 (≤5 cm) tumors. Of the cohort, 81% were N0, and 19% were N+ tumors. 15% of the patients had G1 tumors, 69% were G2, and 16% had G3 tumors. 91% of patients were ER+/PR+, 8% were ER+/PR-, and only 1 patient tested ER-/PR+. 98% of the patients were HER2- only, 1% of the patients were HER2 equivocal by FISH, and 1 patient tested positive for HER2. 4% of the patients expressed low Ki67 (≤5%), 37% as intermediate Ki67 (6%-29%), and 25% had high Ki67 (≥30%) (Table 1).

**Table 1:** Patient demographics and tumor characteristics CAB: CanAssist Breast, LR: Low Risk, HR: High Risk, ^^ Ki67 data not available for 101 (34%) patients.

Clinicopathological Features	Subgroups	No. of Patients n (%)
	Total	300 (100%)
Age (Years)	≤ 48	67 (22%)
	> 48	233 (78%)
	Median Age	59
Tumor size	T1	98 (33%)
	T2	189 (63%)
	T3	12 (4%)
	T4	1
Node status	N0	242 (81%)
	N+	58 (19%)
Histological Grade	G1	46 (15%)
	G2	206 (69%)
	G3	48 (16%)
Hormone receptor status	ER+/PR+	274 (91%)
	ER+/PR-	25 (8%)
	ER-/PR+	1
	HER2/neu-	295 (98%)
	HER2/neu Equivocal by IHC	4 (1%)
	HER/neu+	1
^^Ki67	≤5 (Low	12 (4%)
	6-29 (Intermediate)	111 (37%)
	≥30(High)	76 (25%)

Risk proportion based on clinical risk assessment and prognostic risk assessment

**Figure 1:**
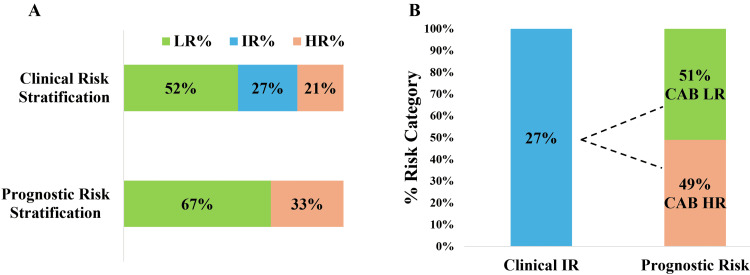
Clinical and CAB risk proportions. B. Re-stratification of clinical intermediate risk category risk. CAB: CanAssist Breast, LR: Low Risk, HR: High Risk, IR: Intermediate Risk.

As shown in Figure 1A, the risk proportions as percentages and numbers based on clinical risk assessment were 52%:27%:21% and 157:80:63 (LR:IR:HR), respectively. Upon prognostication by CAB, the risk proportions were in percentage and numbers as 67%:33% and 201:99 (LR:HR), respectively.

Re-stratification of Clinical Intermediate Risk Category: As shown in Figure 1B, 80 (27%) patients in the clinical IR risk category were re-stratified by CAB as 41 (51%) LR and 39 (49%) HR.

Table 2 presents a detailed overview of the re-stratification of clinical risk proportions based on the CAB irrespective of follow-up data available.

**Table 2:** Re-Stratification of clinical risk proportions based on CAB. CAB: CanAssist Breast, LR: Low Risk, HR: High Risk, IR: Intermediate Risk.

Risk category	Clinical Risk Assessment			
	Clinical LR	Clinical IR	Clinical HR	Total
CAB LR (n)	129	41	31	201
CAB HR (n)	28	39	32	99
Total (n)	157	80	63	300

Drivers of clinical risk assessment

Amongst the various parameters tested, the major drivers of clinical risk assessment were node status, histological grade, patients’ age, and proliferation marker (Ki67) expression status. Low clinical risk is determined mainly based on N0 status, low histological grade, patient age (> 48 years), and low Ki67. Indeed, the majority, 55% (142/242), both T1 63% (63/98) and T2 48% (90/188) tumors are N0, 72% (33/46) and 54% (111/206) G1 and G2, respectively; 55% (128/233) of patients are age (> 48 years old, and 75% (9/12) of low Ki67 patients are clinically LR.

In clinically HR patients, N+ tumors, histological grade 3, age ≤ 48 years at the time of diagnosis, and high Ki67 are perceived to be high-risk features for prescribing chemotherapy. As expected, 38% (4/13) of T3 tumors, 45% (26/58) N+, 31% (15/48) G3, and 30% (23/76) of patients have high Ki67, but only 27% (18/67) of patients with age ≤ 48 years are clinically HR. This may be due to other factors such as node positivity or tumor size outweighing the clinical risk assessment decisions (Table 3).

**Table 3:** Drivers of clinical risk assessment. LR: Low Risk, HR: High Risk, IR: Intermediate Risk.

	Clinical risk assessment (% of patients)		
					LR n (%)	IR n (%)	HR n (%)
Total	157 (52)	80 (27)	63 (21)
≤ 48 (years)	29 (43)	20 (30)	18 (27)
> 48 (years)	128 (55)	60 (26)	45 (19)
N0	142 (59)	63 (26)	37 (15)
N+	15 (26)	17 (29)	26 (45)
T1	62 (63)	18 (18)	18 (18)
T2	91 (48)	58 (32)	40 (20)
T3/T4	4 (31)	4 (31)	5 (38)
G1	33 (72)	8 (17)	5 (11)
G2	111 (54)	52 (25)	43 (21)
G3	13 (27)	20 (42)	15 (31)
Low Ki67	9 (75)	2 (17)	1 (8)
Intermediate Ki67	76 (63)	15 (12)	30 (25)
High Ki67	20 (26)	33 (43)	23 (30)

Patients with a combination of one or more LR and HR features make physicians’ decision to prescribe chemotherapy more complicated, thereby putting these patients in the ‘IR’ category. For example, older patients with N0, with higher grades (G2, G3) (Table 3).

Treatment recommendations based on CAB

**Figure 2:**
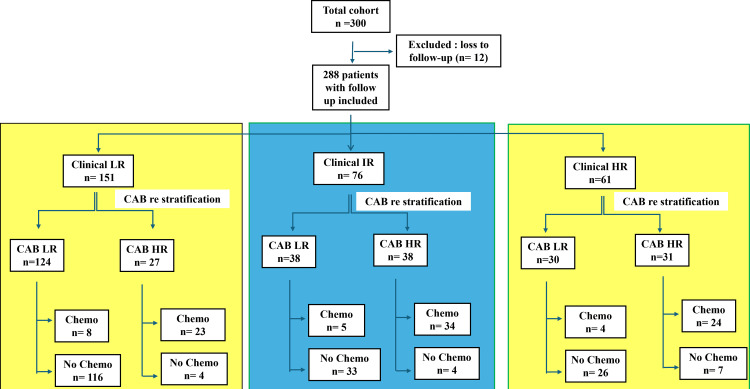
The adaptation of treatment recommendations based on CAB: yellow highlights the change in treatment recommendations, and blue highlights help in treatment decisions. CAB: CanAssist Breast, LR: Low Risk, HR: High Risk, IR: Intermediate Risk, Chemo: chemotherapy.

Out of 300 patients, only 288 follow-up data were available. Among 151 clinical LR patients with follow-up data, 27 patients were CAB HR, and in 23 (85%), there was a change in treatment plan from no chemotherapy to chemotherapy. Similarly, out of 61 clinical HR patients, 30 patients were CAB LR, and in 26 (87%) patients, there was a change from chemotherapy to no chemotherapy. In 76 clinical IR patients, we were able to decide on treatment, as 33 out of 38 CAB LR patients (87%) were not overtreated with chemotherapy, and 34 out of the 38 CAB HR patients (89%) were given chemotherapy. CAB has changed treatment recommendations in 23% (49/212) of the cohort, and for 88% (67/76) of clinical IR patients, CAB helped us to make a treatment decision. Any deviation from the treatment recommendation by CAB is due to other clinical and family history factors (Figure 2).

Treatment adherence with CAB results

Out of 288 patients with follow-up data available, 91% of CAB LR patients were not treated with chemotherapy, and 84% of CAB HR patients were treated with chemotherapy. In 32 (11%) patients, the physicians did not adhere to the CAB results. Among them, 9% of CAB LR patients were treated with chemotherapy, and 16% of CAB HR were not given chemotherapy (Table 4).

**Table 4:** Adherence by physician to CAB test results. CAB: CanAssist Breast, LR: Low Risk, HR: High Risk.

	CAB LR n (%)	CAB HR n (%)
Total (n= 288)	192 (67)	96 (33)
No Chemotherapy	175 (91)	15 (16)
Chemotherapy	17 (9)	81 (84)
Treatment concordance (%)	91%	84%

## Discussion

Prognostic tests have shown that proliferation markers and other biological features that reflect aggressive tumor biology and cancer relapse are accurate determinants of clinical outcomes [10-15], [21-23]. Among the multiple prognostic tests currently available for patients, CAB is the most recent modality on the block. Unlike multi-gene tests (Oncotype DX, MammaPrint, EndoPredict, Prosigna), which examine gene expression, CanAssist Breast assesses protein expression using immunohistochemistry techniques [10-12], [21], [28, 29]. Traditionally, clinicians have used IHC-based methods (for ER, PR, and HER2 expression) along with clinical parameters to assess the recurrence risk and plan the treatment. IHC4 is one such online prognostic test that uses immunohistochemical grading of ER, PR, HER2, and Ki-67 to predict cancer relapse. IHC4 was developed in the TransATAC cohort and showed equivalent performance with multi-gene tests [28,30]. These comparative studies show that prognostication is independent of the technique used and could be either IHC-based or genomics-based. Recently, CAB has been validated in a sub-cohort of TEAM trial patients [31]. CAB is included in the Asian Geriatric Oncology Society’s guidelines as well [32]. An opinion poll on treating HR+/HER2− EBC from 185 Indian oncologists from low- to medium-income countries, in which the utility of CAB was mentioned. [33]. The current study aims to compare the risk assessment made by physicians solely based on an understanding of the clinicopathological features to that of the CAB test, which uses five additional biomarkers for risk assessment. In addition, we report the impact of CAB on changes in physicians’ treatment decisions.

While the clinician's risk assessment is based on age, tumor size, grade, lymph node status, and Ki67, the CAB risk assessment is primarily based on underlying tumor biology in addition to tumor size, grade, and node status. Significantly more patients were classified as LR by CAB than by us, showing a discrepancy in risk assessment between CAB and clinical judgment. Importantly, CAB was proven to be useful in assigning a risk category to patients with ambiguous clinical IR categories. The uncertainty of prescribing chemotherapy in the clinical IR category is primarily due to a combination of low-risk features such as older age/N0 disease/low Ki67 coupled with one or more HR features such as young age/N+ disease/high Ki67. In this study, 34% of Ki67 data is not available because of varying cut-off values, inter-observer variability, and grading criteria; therefore, many hospitals in India unfortunately do not routinely test for Ki67.

In the current cohort, notable changes were seen across the cohort after CAB re-stratification, reflecting a shift in treatment recommendations both in clinically low- and high-risk groups. CAB, which emphasizes tumor biological characteristics to determine disease aggressiveness, offers clear and precise treatment options for patients.

The strengths of this study were the patient data from several oncology centers across India, and the major limitation is presenting data on only 300 patients from our centers combined. It would be interesting to assess the clinical outcomes of these patients. after the completion of five years of follow-up (by the end of 2026). We look forward to publishing that data, which would be a particularly useful validation of CAB, in this prospective cohort.

## Conclusions

In conclusion, our data depicts how CAB is useful in guiding physicians in chemotherapy treatment planning and thereby supporting the clinical utility of this relatively newer test. CAB stratifies more patients as LR and spares them from chemotherapy. CAB also helps physicians stratify IR patients for whom making a treatment decision is very complicated without the help of a prognostic test. With the extensive analytical validation and clinical validation data in patients across geographies, we believe that CAB will be useful in tailoring chemotherapy decisions in clinical practice.
